# Regulatory mechanisms of miR-212-3p on the secretion of inflammatory factors in monocyte-macrophages and the directed differentiation into osteoclasts in ankylosing spondylitis

**DOI:** 10.18632/aging.205249

**Published:** 2023-11-28

**Authors:** Jianli Xie, Jinrong Xu, Haiying Chen

**Affiliations:** 1 Department of Rheumatic Immunology, The Third Hospital of Hebei Medical University, Shijiazhuang, China

**Keywords:** ankylosing spondylitis, miR-212-3p, monocyte-macrophages, inflammatory factors, osteoclasts

## Abstract

To explore the mechanisms of action of micro ribonucleic acid (miR)-212-3p in the secretion of inflammatory factors in monocyte-macrophages and the directed differentiation into osteoclasts (OCs) in ankylosing spondylitis (AS), proteoglycan was used to establish an AS mouse model. The mouse monocyte-macrophages were cultured *in vitro*, transfected with miR-212-3p mimic, and added with phosphorylated-extracellular signal-regulated kinase (p-ERK)1/2 agonist Ro67-7476 *in vitro*. After the cells were transfected with the miR-212-3p mimic in each group, the expressions of p-ERK1/2, matrix metalloproteinase-1 (MMP-1), MMP-3, interleukin-1β (IL-1β), and tumor necrosis factor-α (TNF-α) significantly declined, whereas those of tartrate-resistant acid phosphatase (TRAP), calcitonin, and p-nuclear factor of activated T cell 1 (NFATC1) significantly rose. After Ro67-7476 was added, the protein expressions of p-ERK1/2, MMP-1, MMP-3, IL-1β, and TNF-α were significantly increased in each group, but they displayed decreasing trends in cells transfected with the miR-212-3p mimic. In contrast, the protein expressions of TRAP, calcitonin, and p-NFATC1 declined, but they showed increasing trends in cells transfected with the miR-212-3p mimic. miR-212-3p can, through inhibiting the phosphorylation of p-ERK1/2, prevent the aggregation of macrophages and the secretion of inflammatory factors. It also up-regulates the expression of OC marker proteins to facilitate the differentiation and maturation of OCs, ultimately relieving AS-induced inflammation and new bone growth-induced joint neoplasm.

## INTRODUCTION

Ankylosing spondylitis (AS) is a chronic autoimmune disease primarily characterized by inflammation and pathological ossification [1]. With the development of the disease, pathological changes manifested as ectopic osteogenesis and bone destruction occur, ultimately leading to severe symptoms such as joint destruction and deformity and spinal stiffness, and greatly affecting the quality of life of patients [2]. Studies have revealed that the pathological inflammation of AS is associated with bacterial infection [3], macrophage activation [4], some cytokines [5], HLA-B27 misfolding [6], and autophagy [7]. Macrophages are one of the cells infiltrated at the pathogenic site of AS, and as the major cells of non-specific immune response, they are also important players in the activation of the inflammatory response, immunoregulation, and inflammatory process of AS [8].

During the development of AS, the loss of joint mobility and the sharp decline in quality of life are mainly caused by joint destruction and new bone formation [9]. New bone growth-induced joint neoplasm is a pathological feature of AS, but AS patients often have low bone mineral density/fractures. Some researchers argue that AS becomes pathologically irreversible once bone destruction occurs [10, 11]. Therefore, controlling the early development of AS is one of the key links in the improvement of clinical prognosis of AS, so preventing the new bone growth-induced joint neoplasm will be an important component of the treatment of AS. Osteoclasts (OCs), multinuclear cells evolving from the monocyte-macrophage lineage, are an important part of the bone coupling [12]. During bone development, the roles of bone formation of osteoblasts and the bone resorption of OCs are tightly coupled and involved in bone homeostasis [13]. A large number of monocyte-macrophages will migrate around the inflammatory joint under the influence of a variety of factors, and differentiate into OCs under the induction of receptor activator of nuclear factor-κB ligand (RANKL) [14]. Extracellular signal-regulated kinase (ERK)1/2, a member of the mitogen-activated protein kinase (MAPK) family, mainly plays a role in signal transmission and can induce a series of different intracellular reactions [15]. Both MAPK and ERK are considered key factors in the differentiation and activation of OCs [16]. It has been found that inhibiting the ERK signaling pathway can enhance the differentiation of OCs and the expressions of OC markers tartrate-resistant acid phosphatase (TRAP), calcitonin receptor, and cathepsin K and nuclear factor of activated T cell 1 (NFATC1) [17].

Micro ribonucleic acids (miRNAs) are a class of non-coding small RNAs recently discovered to be able to regulate gene expression, which can bind to the 3'-untranslated region (3’UTR) of target genes, thereby inhibiting the protein transcription. Previously, it was confirmed that miRNAs are widely involved in osteoblast and OC differentiation [18]. In the present study, it was found through The Cancer Genome Atlas (TCGA) database that miR-212-3p was lowly expressed in AS, and the mechanisms of action of miR-212-3p in the secretion of inflammatory factors in monocyte-macrophages and the directed differentiation into OCs in AS were explored, so as to provide valuable clues for the treatment of AS.

## MATERIALS AND METHODS

### Bioinformatics analysis

The datasets related to AS were searched in the Gene Expression Omnibus (GEO) database (https://www.ncbi.nlm.nih.gov/gds/), and the dataset GSE11886 of gene expressions related to AS was found and downloaded. Besides, the dataset GSE118806 containing miRNA sequencing data of AS patients was downloaded. The quantiles of RNA sequencing (RNA-seq) data were standardized and the differentially expressed genes (DEGs) were analyzed using the R language (The R Foundation of Statistical Computing, Vienna, Austria) Limma package (|logFC|<1, P<0.05). The volcano plot of the dataset GSE11886 for visual grouping of DEGs was constructed in the R software using the ggplot2 package, and a cluster analysis heat map of DEGs was plotted using the pheatmap package of the R software. In addition, a volcano plot of GSE118806 for visual grouping of DEGs and its cluster analysis heat map were constructed using the same methods.

### Functional enrichment analysis

The DEGs in the dataset GSE11886 were subjected to Gene Ontology (GO) and Kyoto Encyclopedia of Genes and Genomes (KEGG) enrichment analyses. The DEGs at the biological process (BP), cellular component (CC), and molecular function (MF) levels were analyzed using the Database for Annotation, Visualization, and Integrated Discovery (DAVID) online database tool (https://david.ncifcrf.gov) to integrate the GO terminology, and a BP network of DEGs was created. The GO pathway map and KEGG pathway enrichment analysis map of DEGs were graphed using the GOplot and ggplot2 packages in R.

### Prediction of miRNA target genes

The miRNA candidate target genes were predicted using the online tools TargetScan (https://www.targetscan.org/vert_80/) and mirDIP, and were combined with the DEGs in GSE118806 to predict the target genes by drawing a Venn diagram using the VennDiagram package. Moreover, the binding sites between messenger RNA (mRNA) and miRNA were plotted according to the gene prediction results.

### Gene set enrichment analysis

Gene set enrichment analysis (GSEA) of all genes was conducted by means of the GSEA tool (http://www.gsea-msigdb.org/), and the GSEA pathway map was graphed.

### Statistical analysis

The DEseq2 and ggpubr packages of R software (v3.6.1) were utilized for statistical analysis. The Wald test was adopted to analyze the DEGs. Besides, rank sum testing was conducted to compare cytokines between 2 groups. A P value <0.05 indicated that the difference was statistically significant.

### Animal feeding and induction of an AS mouse model

A total of 48 healthy male BALB/c mice aged 4–5 weeks were provided by Suzhou Joinn Laboratories Co., Ltd. (Suzhou, China). All mice were housed under pathogen-free conditions and fed with a standard diet, with water ad libitum. After adaptive feeding for 1 week, the BALB/c mouse model (n=24) was induced by proteoglycan following the method described previously [19]. Specifically, 100 μL of proteoglycan (100 μg) and 100 μL of Freund's complete adjuvant (0.85mL of paraffin oil and 0.15mL of mannitol monooleate per mL) were evenly mixed and intraperitoneally injected into the mice 3 times at an interval of 3 weeks. At 4 weeks after the last injection, the AS mouse model was established successfully under the following criteria: polyarticular synovitis, midarticular deformity or stiffness, and pathological changes accompanied by the proliferation of chondrocytes and mononuclear cell infiltration in the annulus fibrosus. Meanwhile, mice in the normal control group (n=24) received the same dose of physiological saline. All mice were sacrificed under 50 mg/kg of sodium pentobarbital after 4 weeks of injection.

### Isolation and culture of monocyte-macrophages

Peripheral venous blood was aseptically drawn from mice, mixed evenly with phosphate-buffered saline (PBS), added with lymphocyte separation medium and centrifuged at 2,000 r/min for 20 minutes, and the lymphocyte layer was aspirated. The cells were washed twice with PBS, centrifuged at 5,000 r/min for 10 minutes, added with 10% fetal bovine serum-containing Roswell Park Memorial Institute (RPMI) 1640 medium preheated to 37° C, pipetted evenly and incubated in a 5% CO_2_ incubator at 37° C for 4–7 days, and the medium was replaced every 48 hours. Then, the monocyte-macrophages isolated and cultured were inoculated into a plate at 1×10^6^ cells/well, and added with 40 ng/mL macrophage colony stimulating factors (M-CSFs) to promote the differentiation and adherence of macrophages.

### Cell transfection and grouping

The well-growing normal and AS cells were transfected with miR-212-3p mimic using Lipofectamine 2000 (Thermo Fisher, Waltham, MA, USA), and the p-ERK1/2 agonist Ro67-7476 was added into the medium in each group. Finally, the macrophages were divided into 8 groups, as follows: normal group (macrophages from control mice), AS group (macrophages from AS mice), normal+miR-212-3p mimic group (macrophages from control mice+miR-212-3p mimic), AS+miR-212-3p mimic group (macrophages from AS mice+miR-212-3p mimic), normal+Ro67-7476 group (macrophages from control mice+Ro67-7476), AS+Ro67-7476 group (macrophages from AS mice+Ro67-7476), normal+miR-212-3p mimic+Ro67-7476 group (macrophages from control mice+miR-212-3p mimic+Ro67-7476), and AS+miR-212-3p mimic+Ro67-7476 group (macrophages from AS mice+miR-212-3p mimic+Ro67-7476).

### Western blotting

The protein was extracted from cells using protein extraction kits, and its concentration was measured. Then, 50 μg of protein was taken and subjected to sodium dodecyl sulfate polyacrylamide gel electrophoresis (SDS-PAGE), transferred onto a nitrocellulose membrane using point transfer method, sealed with 3.0% skim milk powder, and incubated with p-ERK1/2, t-ERK1/2, MMP-1, MMP-3, IL-1β, TNF-α, TRAP, calcitonin, p-NFATC1, and β-actin primary antibodies (1:1000) at 4° C overnight. After the membrane was washed, horseradish peroxidase (HRP)-labeled goat anti-rabbit secondary antibodies (1:5000) were added for incubation at room temperature for 2 hours. After chemiluminescence, the images were acquired and subjected to grayscale analysis using Quantity One software (Bio-Rad, Hercules, CA, USA). The gray levels of the protein to be detected and β-actin were compared and analyzed.

### Statistical analysis

The software SPSS 16.0 (IBM Corp., Chicago, IL, USA) was used for statistical analysis, and the values were expressed as mean ± standard deviation. The means were compared between 2 groups by t-test or Mann-Whitney test for non-parametric parameters. The comparison among groups was performed by one-way analysis of variance (ANOVA) and least significant difference (LSD) pairwise comparison. A P value <0.05 was considered statistically significant.

## RESULTS

### Screening of DEGs

**Figure 1:**
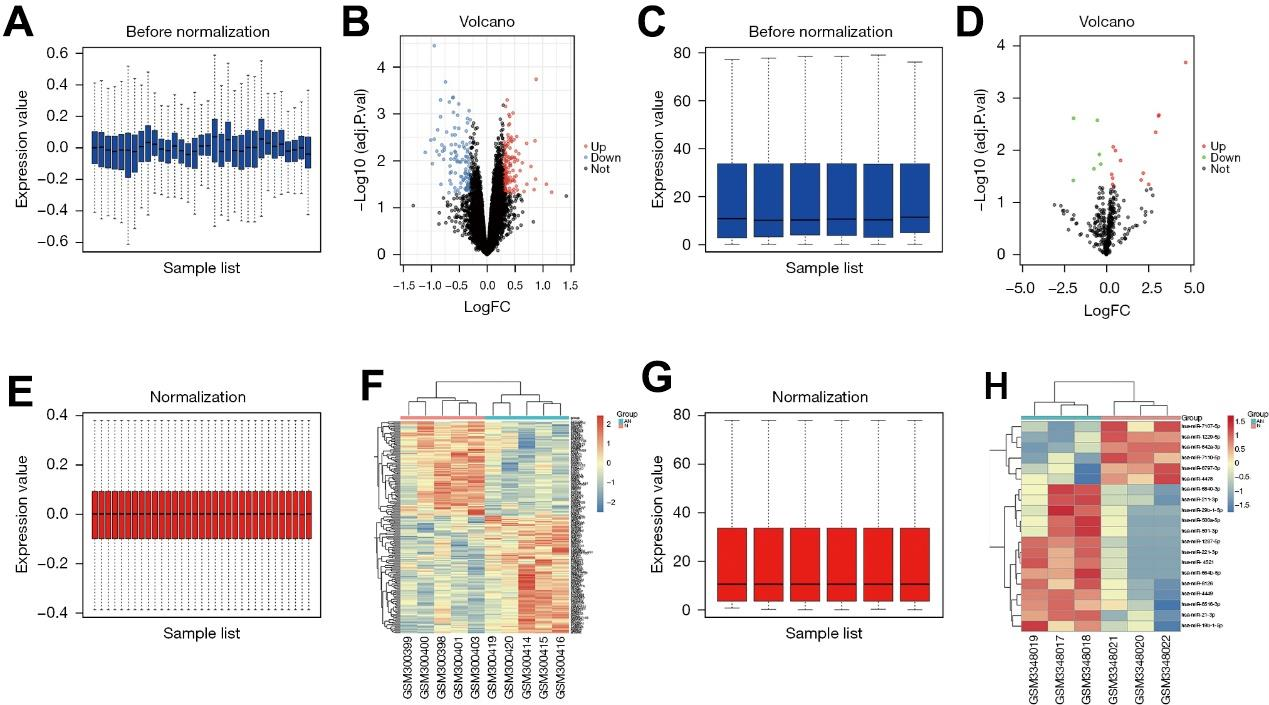
(**A**) GSE11886 pictures of the database prior to the quantitative normalization. (**B**) The GSE11886 dataset was screened according to P<0.05, |logFC|<1. The results showed that there were 263 DEGs in ankylosing spondylitis mRNA, of which 149 were up-regulated and 114 were down-regulated. The volcano map of dataset GSE11886 was constructed in R software using ggplot2 software package. (**C**) Images of GSE118806 data set after standardized processing. (**D**) Volcano map of the GSE118806 dataset. (**E**) Image after quantitative standardization processing of the GSE11886 database. (**F**) Pheatmap software package in R software was used to draw DEGs cluster analysis heatmap. (**G**) Images after standardized processing of the GSE118806 dataset. (**H**) Clustering heat map of the GSE118806 dataset.

The dataset GSE11886 related to AS was downloaded from the GEO database, and the data quantiles were standardized (Figure 1A, 1E). GSE11886 was screened according to the criteria of P<0.05 and |logFC|<1. The results showed that there were 263 DEGs in the mRNAs of AS, of which 149 were up-regulated and 114 were down-regulated. The volcano plot of the dataset GSE11886 for visual grouping of DEGs was constructed in the R software using the ggplot2 package (Figure 1B), and the cluster analysis heat map of DEGs was plotted using the pheatmap package of R software (Figure 1F). In addition, the quantiles of the miRNA dataset GSE118806 were standardized (Figure 1C, 1G), and the volcano plot of GSE118806 for visual grouping of DEGs (Figure 1D) and its cluster analysis heat map (Figure 1H) were constructed using the same methods.

### Bioinformatics analysis

**Figure 2:**
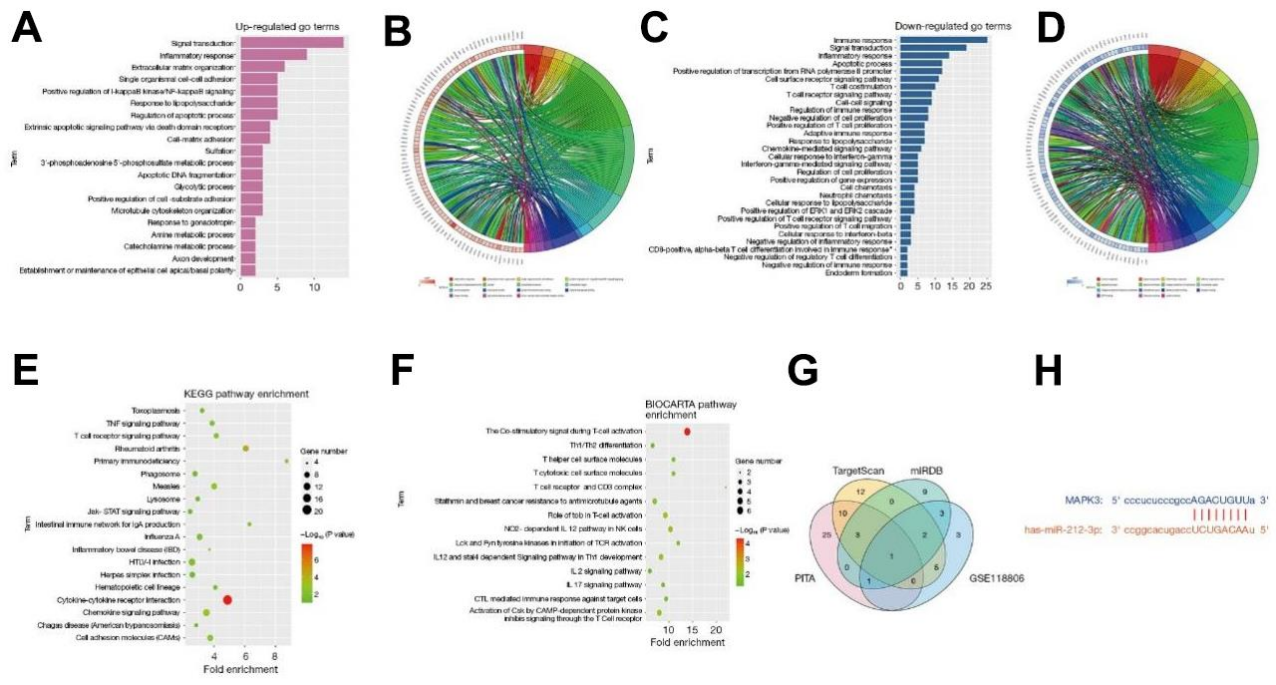
**The DEGs obtained in GSE11886 were analyzed by GO and KEGG enrichment analyses.** The corresponding DEGs at the BP level were analyzed using the DAVID online database tool to integrate the GO terminology, and a BP network of DEGs was created. The up-regulation pathway diagram of GO pathway of DEGs (**A**, **B**) was graphed using the R language, and the up-regulated pathways such as inflammatory response, negative regulation of apoptotic process, and innate immune response were obtained. The down-regulation pathway diagram (**C**, **D**) was graphed, and it was found that the down-regulation pathways such as protein phosphorylation, positive regulation of GTPase activity, and rRNA processing were the enrichment pathways of AS. The DEGs were utilized to analyze the KEGG pathway and a KEGG pathway diagram was drawn (**E**), from which the ERK pathway and other pathways were obtained. Besides, the BioCarta pathway was analyzed, and its pathway diagram was graphed (**F**). The miRNA candidate target genes were predicted using the online tools TargetScan, miRDB, and PITA. Based on these candidate target genes and the DEGs in GSE118806, a Venn diagram was drawn with the VennDiagram package to obtain the intersection and find the common binding miR-212-3p (**G**). The binding sites between mRNA and miRNA were plotted according to the gene prediction results (**H**). All genes were subjected to GSEA using the GSEA tool (http://www.gsea-msigdb.org/). DEGs, differentially expressed genes; BP, biological process; DAVID, Database for Annotation, Visualization, and Integrated Discovery; GO, Gene Ontology; KEGG, Kyoto Encyclopedia of Genes and Genomes; AS, ankylosing spondylitis; ERK, extracellular-signal-regulated kinase; mRNA, messenger RNA; miRNA, microRNA; GSEA, Gene Set Enrichment Analysis.

The DEGs obtained in GSE11886 were analyzed by GO and KEGG enrichment analyses. The corresponding DEGs at the BP level were analyzed using the DAVID online database tool to integrate the GO terminology, and a BP network of DEGs was created. Moreover, the up-regulation pathway diagram of GO pathways of DEGs (Figure 2A, 2B) and their down-regulation pathway diagram (Figure 2C, 2D) were graphed using the R language. As can be seen from the GO pathway diagram, the up-regulated pathways, such as inflammatory response, negative regulation of apoptotic process, and innate immune response, and the down-regulated pathways, such as protein phosphorylation, positive regulation of GTPase activity, and ribosomal RNA (rRNA) processing were the enrichment pathways of AS. Furthermore, the DEGs were utilized to analyze the KEGG pathways and a KEGG pathway diagram was drawn (Figure 2E), from which the ERK pathway and other pathways were obtained. Besides, the BioCarta pathway was analyzed, and its pathway diagram was graphed (Figure 2F).

The miRNA candidate target genes were predicted using the online tools TargetScan, miRDB, and PITA. Based on these candidate target genes and the DEGs in GSE118806, a Venn diagram was drawn with the VennDiagram package to obtain the intersection and find the common binding miR-212-3p (Figure 2G). Moreover, the binding sites between mRNA and miRNA were plotted according to the gene prediction results (Figure 2H).

### Statistical analysis of target genes

**Figure 3:**
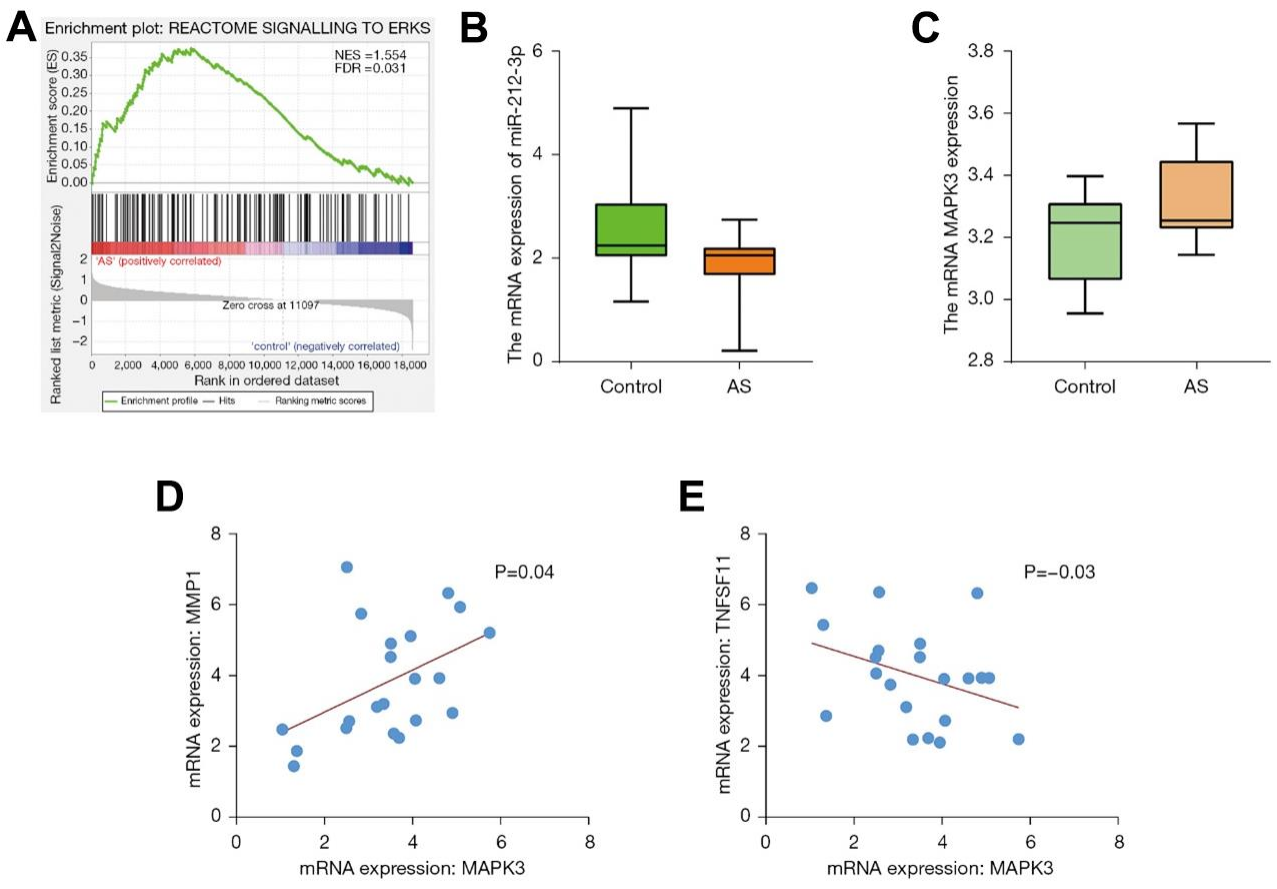
**The GSEA pathway map was graphed.** The results of GSEA manifested that the ERK pathway and other pathways were the enrichment pathways (**A**). The content of genes in different groups was subjected to statistical analysis. miR-212 was lowly expressed in the disease group and highly expressed in the control group (**B**). MAPK3 was highly expressed in the disease group (**C**). The results of correlation analysis revealed that MAPK3 was correlated with MMP (**D**) and TNFSF1 (**E**). GSEA, Gene Set Enrichment Analysis; ERK, extracellular-signal-regulated kinase; MAPK, mitogen-activated protein kinase; MMP, matrix metalloproteinase; TNFSF1, tumor necrosis factor ligand superfamily member 1.

The GSEA analysis manifested that the ERK pathway and other pathways were the enrichment pathways (Figure 3A). The content of genes in different groups was subjected to statistical analysis. It was shown that miR-212 was lowly expressed in the disease group and highly expressed in the control group (Figure 3B). Meanwhile, MAPK3 was highly expressed in the disease group (Figure 3C), and the results of correlation analysis revealed that MAPK3 was correlated with MMP (Figure 3D) and tumor necrosis factor ligand superfamily member 1 (TNFSF1; Figure 3E).

### miR-212-3p mimic inhibited the secretion of inflammatory factors in monocyte-macrophages in early AS by regulating the activation of p-ERK1/2

**Figure 4:**
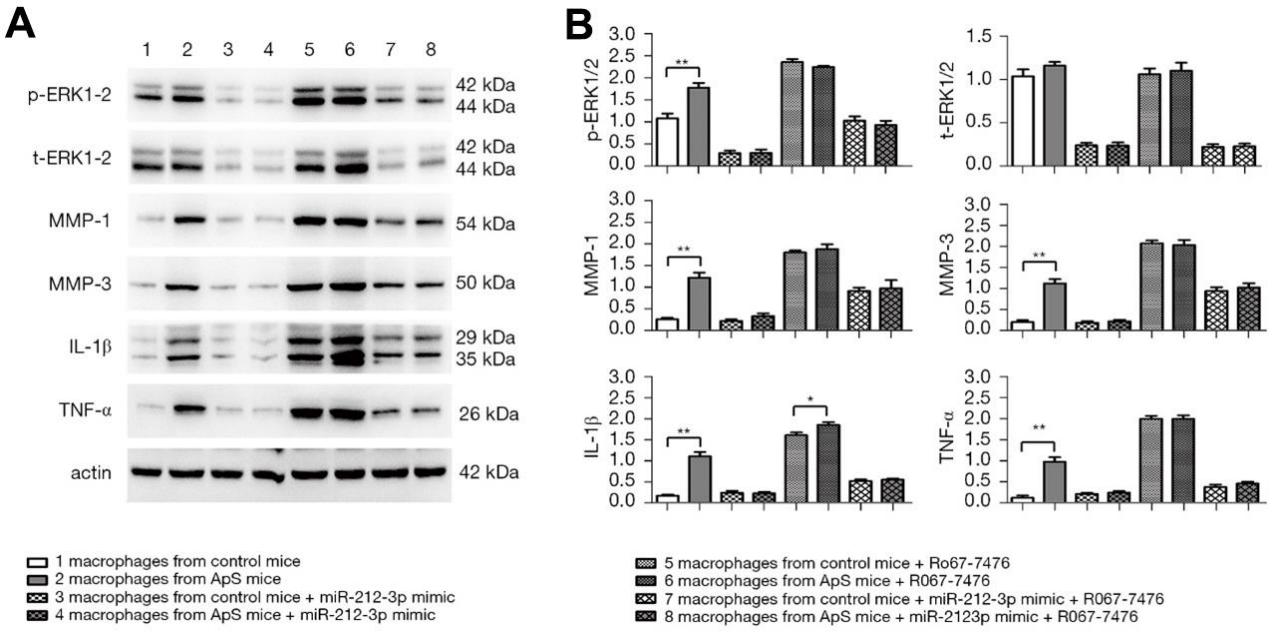
(**A**) Protein expression of p-ERK1/2, t-ERK1/2, MMP-1, MMP-3, IL-1β and TNF-α in monocyte-macrophage cells was detected by Western blot (ERK: extracellular signal-regulated kinase; MMP: matrix metalloproteinase; IL-1β: interleukin-1β; TNF-α: tumor necrosis factor α). (**B**) Statistical histogram of relative protein expression.

The protein expressions of p-ERK1/2, t-ERK1/2, MMP-1, MMP-3, IL-1β, and TNF-α in monocyte-macrophages were measured by western blotting (Figure 4A, 4B). The results showed that the protein expressions of p-ERK1/2, MMP-1, MMP-3, IL-1β, and TNF-α significantly rose in AS-derived monocyte-macrophages compared with those in monocyte-macrophages in normal mice. The factors all significantly declined after monocyte-macrophages from AS mice were transfected with miR-212-3p mimic. After Ro67-7476 was added, the protein expressions of p-ERK1/2, MMP-1, MMP-3, IL-1β, and TNF-α were significantly increased in each group, but they displayed decreasing trends in cells transfected with miR-212-3p mimic. The above findings suggest that miR-212-3p mimic can suppress the protein expressions of MMP-1, MMP-3, IL-1β, and TNF-α in monocyte-macrophages in AS through regulating the activation of ERK1/2.

### miR-212-3p mimic promoted the directed differentiation of monocyte-macrophages into OCs in early AS by regulating p-ERK1/2

**Figure 5:**
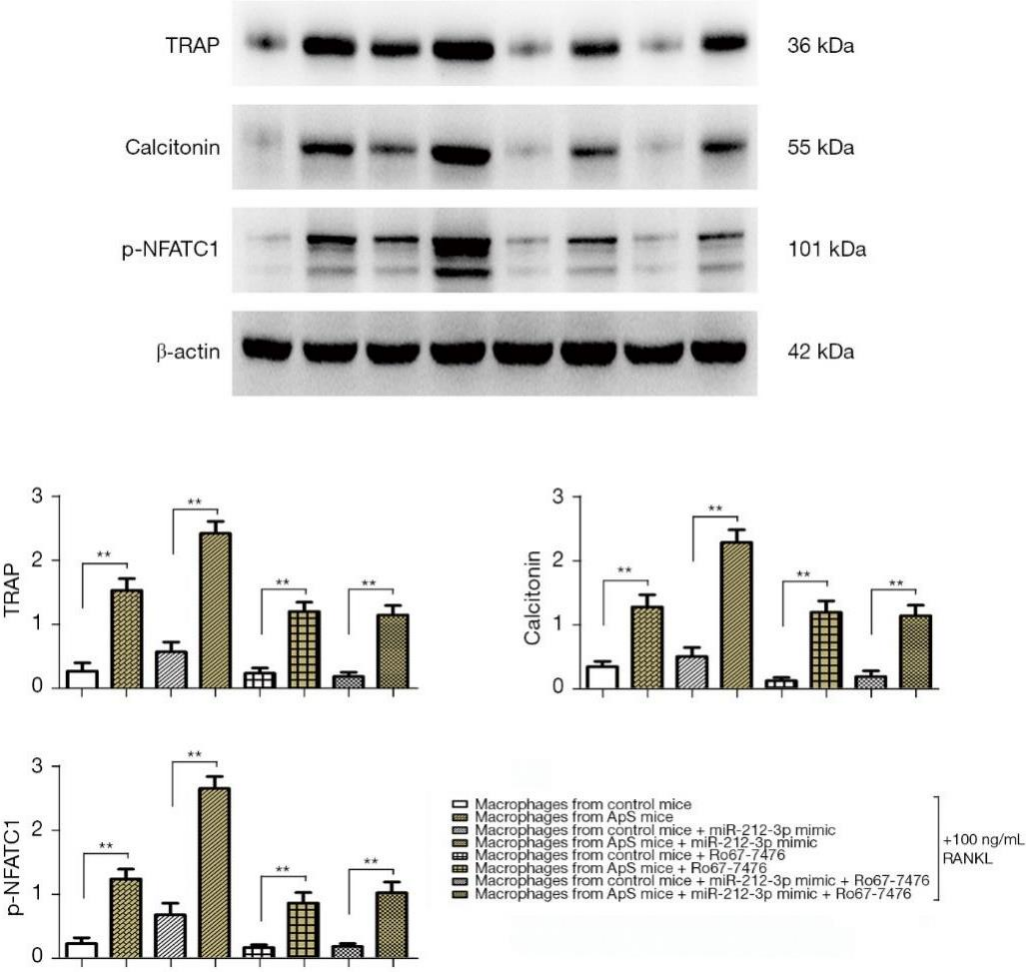
**The directed differentiation of monocyte-macrophages into OCs was induced by 100 ng/mL RANKL added to the medium in each group, and the protein expressions of TRAP, calcitonin, and p-NFATC1 in monocyte-macrophages were measured by Western blotting.** OCs, osteoclasts; RANKL, receptor activator of nuclear factor-κB ligand; TRAP, tartrate-resistant acid phosphatase; NFATC1, nuclear factor of activated T cell 1.

The directed differentiation of monocyte-macrophages into OCs was induced by 100 ng/mL RANKL added to the medium in each group. The results showed that the protein expressions of TRAP, calcitonin, and p-NFATC1 significantly increased in AS-derived monocyte-macrophages compared with those in monocyte-macrophages of normal mice. They all significantly rose after the cells were transfected with miR-212-3p mimic, and the factors all significantly rose in monocyte-macrophages from AS mice were transfected with miR-212-3p mimic, compared with monocyte-macrophages in normal mice were transfected with miR-212-3p mimic (Figure 5A, 5B). It can be inferred that the overexpression of miR-212-3p can facilitate the maturation and differentiation of monocyte-macrophages into OCs in AS. After Ro67-7476 was added, the protein expressions of TRAP, calcitonin, and p-NFATC1 declined, and the protein expressions of OC markers were up-regulated in monocyte-macrophages from AS mice compared with those in monocyte-macrophages of normal mice. The above findings indicate that miR-212-3p mimic may promote the directed differentiation of monocyte-macrophages into OCs in early AS by inhibiting the activation of p-ERK1/2.

## DISCUSSION

**Figure 6:**
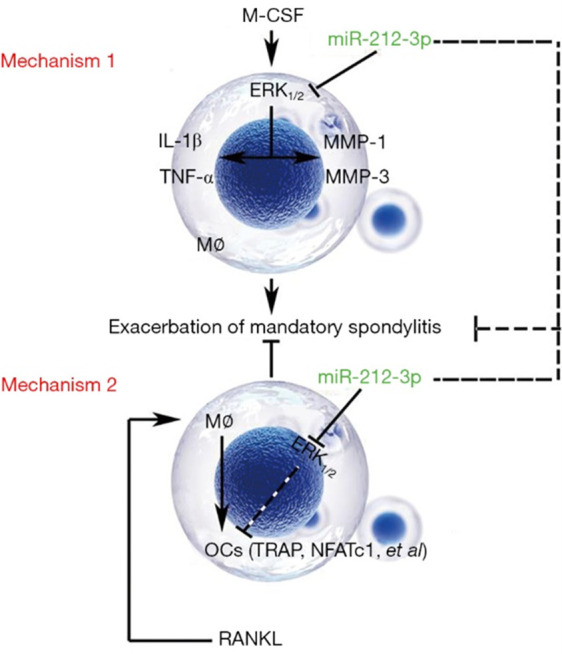
**Regulatory mechanisms of miR-212-3p on the secretion of inflammatory factors in monocyte-macrophages and the directed differentiation into OCs in AS.** Mechanism 1: miR-212-3p inhibits the protein expressions of MMP-1, MMP-3, IL-1β and TNF-α through suppressing the activation of p-ERK1/2, thereby preventing the aggregation of macrophages and the secretion of inflammatory factors in AS. Mechanism 2: miR-212-3p mimic promotes the directed differentiation of monocyte-macrophages into OCs in AS through inhibiting p-ERK1/2 (RANKL: It induces the differentiation of monocyte-macrophages into OCs). M-CSF, macrophage colony stimulating factor; ERK, extracellular signal-regulated kinase; MMP, matrix metalloproteinase; IL-1β, interleukin-1β; TNF-α, tumor necrosis factor-α; OCs, osteoclasts; AS, ankylosing spondylitis; TRAP, tartrate-resistant acid phosphatase; NFATC1, nuclear factor of activated T cell 1; RANKL, receptor activator of nuclear factor-κB ligand.

As a chronic spondyloarthritis, AS is primarily characterized by inflammation and pathological ossification [20]. Some studies have revealed that the pathological inflammation of AS has an association with bacterial infection (3), macrophage activation (4), some cytokines (5), HLA-B27 misfolding (6), and autophagy (7). Macrophages are one of the cells infiltrated at the pathogenic site of AS, and as the major cells of non-specific immune response, they are also important players in the activation of inflammatory response, immunoregulation, and inflammatory process of AS (8). In the present study, a proteoglycan-induced AS mouse model was established, and the monocyte-macrophages were cultured *in vitro*. The results showed that the protein expressions of IL-1β and TNF-α in monocyte-macrophages in AS mice were significantly higher than those in normal mice, and miR-212-3p mimic could inhibit the protein expressions of IL-1β and TNF-α in monocyte-macrophages in AS through regulating the activation of ERK1/2, thereby reducing the secretion of inflammatory factors (Figure 6). Monocytes in blood and bone marrow can be induced to differentiate into OCs with bone resorption activity [21]. However, whether such monocytes induced are specific remains ambiguous yet. The maturation process of such monocytes probably covers the migration and differentiation of OC precursors (cells in each stage before OC differentiation and maturation), and formation of mature OCs with bone resorption activity [22]. In this experiment, miR-212-3p mimic could down-regulate the protein expressions of MMP-1 and MMP-3 in monocyte-macrophages via suppressing the activation of ERK1/2, indicating that miR-212-3p can inhibit the aggregation and migration of macrophages, so that the bone resorption and the maturation and differentiation of OCs may be affected.

In recent studies, osteoporosis or bone loss has been found to be a common presentation in AS, with an incidence rate of 50–92% [23, 24], which can occur in the early stage of the disease [25], increasing the risk of fractures and spinal deformity in patients and seriously affecting the prognosis. In addition, AS is characterized by new bone growth-induced joint neoplasm growth [13]. The formation of OCs in AS patients has been rarely studied, and remains controversial. Most researchers believe that OCs are important players in bone destruction. RANKL, RANK, and osteoprotegerin are recently discovered key factors that regulate the differentiation and maturation of OCs, which have been confirmed to play important roles in bone destructive diseases [26, 27]. After maturation, TRAP, a marker for OCs, will be expressed on the surface of OCs [28], indicating that the OCs have had the ability of bone resorption. Therefore, the expression of TRAP can reflect the number of OCs, and also the bone resorption activity *in vivo*. In this study, the directed differentiation of monocyte-macrophages into OCs was induced by 100 ng/mL RANKL added to the medium in each group. The results of western blotting revealed that the protein expressions of markers for OCs (TRAP, calcitonin, and p-NFATC1) in the AS group were significantly higher than those in the control group. Their expressions were significantly up-regulated after exogenous overexpression of miR-212-3p, suggesting that miR-212-3p can promote the directed differentiation of monocyte-macrophages into OCs and also ameliorate the new bone growth-induced joint neoplasm in AS (Figure 6). It has been found that inhibiting the ERK signaling pathway can enhance the differentiation of OCs and the expressions of OC markers TRAP, calcitonin receptor, cathepsin K, and NFATC1 [17, 29]. Therefore, 100 ng/mL RANKL was added to the medium in each group to induce the differentiation of monocyte-macrophages into OCs. The protein expressions of TRAP, calcitonin, and p-NFATC1 were all significantly down-regulated after Ro67-7476 was added, suggesting that activated p-ERK1/2 will inhibit the maturation of OCs. It was observed that miR-212-3p can enhance the expressions of OC markers and the differentiation and maturation of OCs through inhibiting the phosphorylation of p-ERK1/2, ultimately ameliorating the new bone growth-induced joint neoplasm in AS.

## CONCLUSIONS

In conclusion, miR-212-3p can suppress the phosphorylation of p-ERK1/2 to prevent the aggregation of macrophages and the secretion of inflammatory factors. It also up-regulates the expression of OC marker proteins to facilitate the differentiation and maturation of OCs, ultimately relieving AS-induced inflammation and new bone growth-induced joint neoplasm.
